# Study on the influence of fracture geometric characteristics on the microscopic crack propagation and energy release mechanism in granite

**DOI:** 10.1038/s41598-025-19103-6

**Published:** 2025-10-09

**Authors:** Tianzhuo Qin, Lianhui Li, Changyong Nie, Jiawei Zhou, Qiuzhuo Liu, Liang Cheng

**Affiliations:** 1 Chongqing Yuxiang Double-line Expressway Co. Ltd., Chongqing, 401,120 P.R. China; 2 China Merchants Chongqing Communications Technology Research & Design Institute Co., Ltd., Chongqing, 400,067 P.R. China

**Keywords:** Granite, Particle flow, Microcrack propagation, Energy release mechanism, Constitutive model, Supercapacitors, Crude oil, Civil engineering

## Abstract

Rockburst is a major safety risk in deep tunnel engineering, closely related to the propagation of fractures and energy release in rock masses. Therefore, a thorough understanding of the laws governing fracture propagation, energy release mechanisms, and their intrinsic relationship with rock failure modes is crucial for the prevention and control of high-energy events. This study, based on the geological characteristics of granite, utilizes PFC2D to establish a numerical model and calibrates the mesoscopic parameters using the trial-and-error method. The mechanical response and energy damage evolution characteristics of fractured granite under different fracture inclinations and lengths are systematically investigated. The results indicate that the inclination and length of fractures significantly affect the failure mode, energy release process, and the extent of crack development in the rock. The failure mechanism is mainly characterized by a shear-dominated, tension-assisted oblique shear-tensile failure mode. When the fracture inclination is 0°, granite is most prone to fracture. As the fracture length increases, the number of macroscopic cracks and the degree of failure reach their maximum when the fracture inclination gradually decreases (60°-30°-15°). A smaller *YU* value corresponds to greater energy release, an increased number of macroscopic cracks, and a higher degree of failure, which is more conducive to crack formation. A damage constitutive model for granite based on dissipated energy density is established, and the analysis shows good agreement between the theoretical and experimental curves. The results provide a theoretical basis for understanding rock mass failure and safety issues during deep tunnel excavation.

## Introduction

Underground water-sealed petroleum storage caverns are advantageous as they feature high safety, low environmental impact, good thermal insulation, long—term storage stability and efficient space—saving, making them suitable for strategic petroleum reserves and sustainable development. In recent years, underground water-sealed petroleum storage caverns have been rapidly developing and constructing in the underground deep space, resulting in complex and variable engineering geological conditions in China. In addition, after large-scale geological tectonic movements, natural deep rock masses have a large number of defects such as joints and fractures inside the rocks, making the mechanical behavior and damage evolution process of rocks show complexity and anisotropy. After excavation, the stress concentration effect occurs, and the original fractures inside the rock begin to initiate, crack, propagate, and penetrate, resulting in rock damage and instability failure, which seriously affects the stability and safety of underground structures^1–3^. Therefore, it is of great significance to deeply explore the influence of fracture geometric characteristics on the law of rock fracture propagation and energy release mechanism for the excavation and prevention of underground water-sealed petroleum storage caverns.

The scholars have carried out a series of studies on the law of rock mass fracture propagation and the law of rock mass failure and damage evolution through laboratory tests and numerical simulations. Scholars have carried out laboratory uniaxial compression tests^4–7^. Brace et al.^4^ found that cracks arranged in a regular pattern can propagate under stress conditions much lower than those required for isolated cracks, providing new insights into the evolution mechanisms of complex crack networks in rock masses. Wong et al.^5^, through uniaxial compression tests on marble and gypsum models, first fully revealed the temporal sequence of initiation and propagation of different types of cracks. Importantly, this resolved the long-standing issue of comparability between different studies caused by inconsistent classification standards. Yang et al.^6^ further elucidated the spatiotemporal evolution of crack propagation at the tip of a single fracture, establishing a classification system comprising nine basic modes, including tensile, shear, and lateral propagation, thus providing new theoretical support for the analysis of mechanisms underlying engineering geological disasters such as slope instability and rockburst. Traditional theories are mostly based on the assumption of closed defects, making it difficult to accurately describe the mechanical behavior of open-type defects commonly found in actual engineering. Lin et al.^7^ conducted systematic theoretical analysis and experimental validation, deriving for the first time a quantitative relationship between the initiation angle of open-type defects and the inclination of prefabricated defects, thereby improving the initiation criterion theory for defects. Conventional uniaxial or triaxial tests cannot truly reflect the fracture behavior of fractured rock masses under complex stress environments; under uniaxial compression, rocks exhibit single crack propagation, while confining pressure in triaxial compression significantly alters the failure mode (e.g., formation of shear bands). Li et al.^8^ conducted true triaxial multi-stress path loading tests on fractured sandstone, established a qualitative correspondence between the multi-scale morphological characteristics of fracture surfaces and failure mechanisms, proposed a quantitative characterization method for failure mechanisms based on fractal theory, and finally elucidated the control laws of *σ*_3_, *σ*_2_, and prefabricated fractures on failure modes. Although previous studies have recognized the influence of fracture geometric characteristics (such as inclination and position) and confining pressure on the mechanical behavior of rock masses, there remain key issues to be resolved regarding the coupling mechanisms of these factors in double-layer composite rock masses. Xiao et al.^9^ designed a limestone-sandstone double-layer composite specimen with prefabricated fractures, revealing the evolution characteristics of strength parameters (peak strength, elastic modulus) and the main controlling factors of fracture-induced rock mass deterioration under varying confining pressure gradients. Columnar jointed rock masses (CJRMs), as a typical anisotropic geological material, have excavation unloading response characteristics that are directly related to the stability of surrounding rock in underground engineering. Que^10^ prepared five types of irregular columnar jointed rock mass specimens with different inclinations, simulated the excavation unloading process using a true triaxial loading–unloading system, and proposed quantitative indicators for anisotropic deterioration. Xue et al.^11^ fabricated novel stainless steel bolts using 3D printing technology and conducted reinforcement tests on rock-like specimens containing primary joints at 15°, 30°, 45°, 60°, and 75° angles. During the experiments, they innovatively employed a hybrid monitoring system integrating acoustic emission (AE) and digital image correlation (DIC) techniques, achieving synchronous and precise tracking of dynamic crack propagation and full-field strain evolution.

Although the above experimental studies have revealed the mechanical responses (such as strength degradation and energy evolution) of fractured rock masses under loading and unloading conditions, limitations due to specimen scale effects and monitoring resolution make it difficult to fully capture the mesoscopic mechanisms of fracture network evolution. To address this, some researchers have used numerical simulation methods to compensate for experimental shortcomings, especially particle flow software. The Parallel Bond Model (PBM) simulates the progressive fracture process of bonded contacts under tensile/shear stress (accompanied by stiffness degradation), more realistically reproducing the brittle failure behavior of rock-like materials^12^. Zhang et al.^13^ conducted numerical uniaxial compression tests on rock masses with different fracture inclinations and lengths using PFC2D. YUAN et al.^14^ established a numerical model of fractured rock under uniaxial compression using PFC2D, revealing the macroscopic mechanical behavior mechanisms during crack initiation, propagation, and microscopic failure in rocks containing single fractures. Aliabadian et al.^15^ systematically evaluated the applicability of 3D printing technology combined with digital image correlation (DIC) and the bonded particle model (BPM) in simulating the true mechanical behavior of rock masses containing pre-existing defects. By designing four typical defect configurations—single-sided, coplanar, partially overlapping, and fully overlapping—they established a comprehensive crack propagation and coalescence analysis framework, providing an innovative research methodology for elucidating the failure mechanisms of complex defective rock masses. Zhou et al.^16^ employed the GPD numerical simulation method to investigate the complete process of crack initiation-propagation-coalescence in rock masses. Based on the Hoek–Brown strength criterion, they developed an elastic-brittle damage constitutive model that accurately characterizes the full damage progression of rock-like materials from microscopic crack evolution to macroscopic failure.

The damage and failure process of rocks is ultimately a process of energy absorption, storage, and release. The energy evolution law during rock deformation and failure has been studied through laboratory experiments and numerical simulations. Existing research mainly focuses on the mechanical response of intact rock masses^17–19^, while systematic understanding of the energy evolution paths and damage accumulation mechanisms in discontinuously fractured rock masses under multi-coupled fields or multiple factors is still lacking. Shuang et al.^20^ compared the macro- and microscopic mechanical behavior differences between fractured and intact rock masses, combined energy transformation characterization and damage variable construction, and revealed the regulatory mechanisms of confining pressure and seepage pressure on the failure modes of fractured rock masses. Zhang et al.^21^ and Ren et al.^22^ respectively studied the energy dissipation characteristics of rocks with different fracture shapes.

Current research mainly focuses on the influence of single fracture parameters (such as length, shape, inclination, etc.) on the macroscopic mechanical behavior of rocks, while studies on the mesoscopic damage evolution and energy-driven mechanisms of rocks under multi-parameter coupling are still insufficient. In particular, there is a lack of systematic understanding of the influence mechanism of the synergistic effect of fracture geometric characteristics on the energy evolution law throughout the rock fracture process. Based on this, this study conducts uniaxial compression tests on granite and constructs a mesoscopic numerical model based on PFC2D, focusing on the influence of the coupling effect of fracture inclination and length on the crack propagation path and energy release characteristics of granite. The instability criterion for rock masses based on strain energy rate proposed in this study can provide quantitative guidance for key construction processes such as optimization of roadway support parameters and selection of roof-cutting and pressure-relief timing, and has important practical value for improving the safety and prevention level of underground engineering.

## Granite model construction

### Laboratory tests

**Fig. 1:**
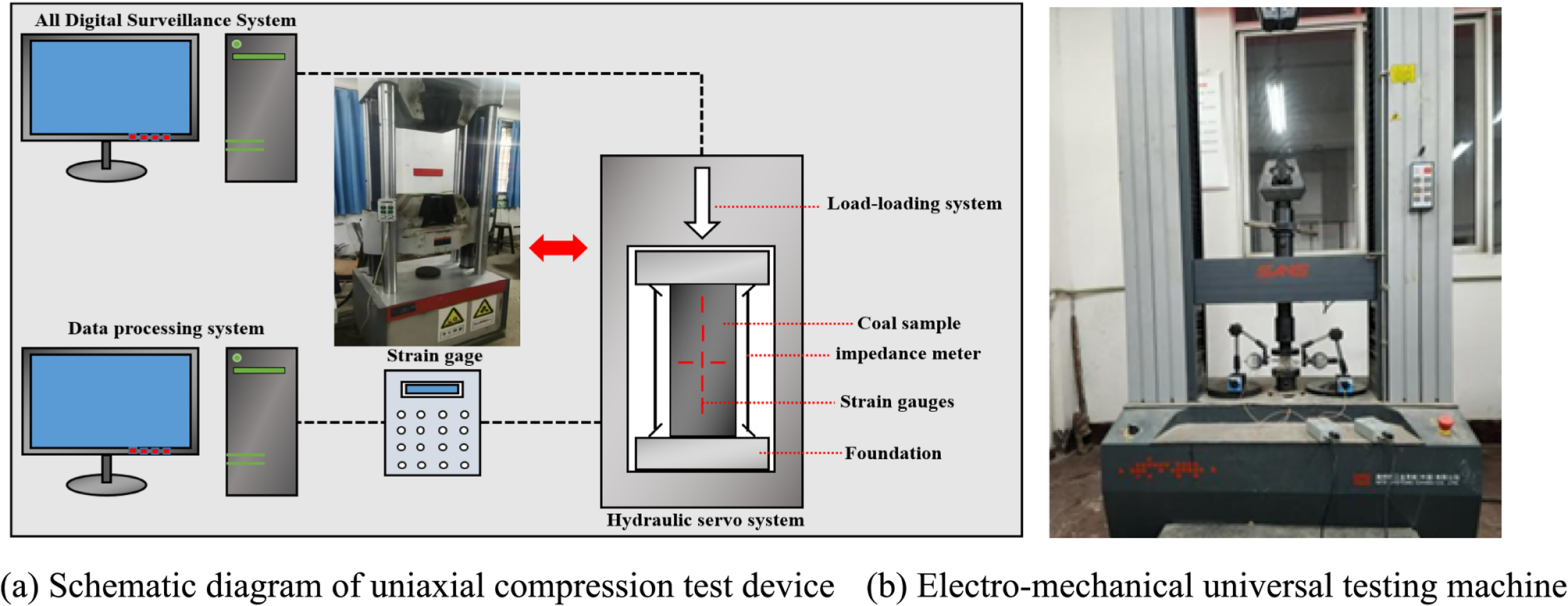
Experimental apparatus.

The specimens in this paper were taken from gneissic granite in a underground water-sealed petroleum storage caverns in Guangdong Province and processed into standard cylinders with a diameter of 50 mm and a height of 100 mm. The natural density is 2730 kg/m^3^, and the main mineral components are mica, amphibole, and pyroxene. In this test, the SANS rock mechanics test system was used to carry out uniaxial compression tests. The displacement loading control method was adopted, and the samples were loaded at a rate of 0.01 mm/s until the samples failed. The stress–strain curve and the final failure mode of granite were obtained. The specific loading device process is shown in Fig. 1.

### Model establishment and mesoscopic parameter verification

The discrete element particle flow (PFC2D) uses the interaction between particles and endows particles with different constitutive models, so it can well simulate different medium samples, especially rock materials. The properties of rock samples established based on PFC2D are highly consistent with the actual rock mechanical properties, revealing the crack initiation, propagation, and failure mechanism of rock tests from a mesoscopic perspective. In this paper, the parallel bond model is adopted. The parallel bond model can simultaneously represent the tensile and compressive stresses of the sample and is often used to simulate materials such as rocks and coal^23,24^.

**Fig. 2:**
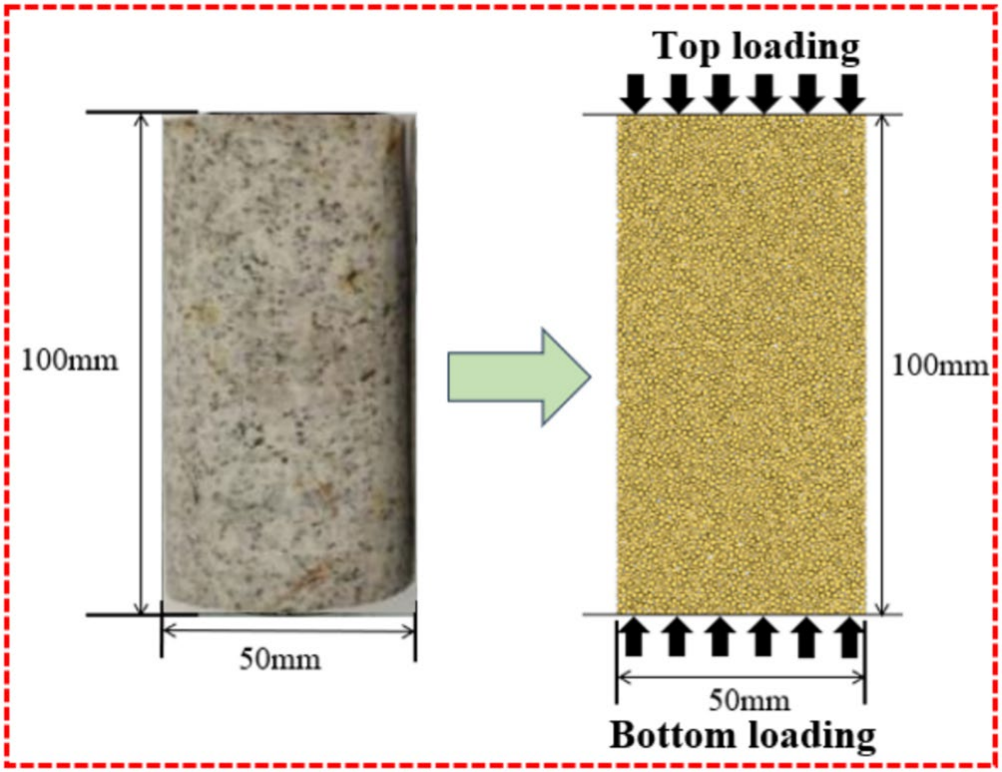
Complete granite samples and numerical model samples.

A standard cylindrical specimen (50 mm × 100 mm) was established using PFC2D, and particles of different sizes were generated and regarded as rigid bodies. Two walls were set on the upper and lower surfaces of the specimen, and a certain speed (0.01 mm/s) was given to the walls to act as loading plates. The correct stop condition was set (the stop condition was 40% of the peak stress), and the specimen failed, that is, the simulation ended. According to the granite laboratory test, the density of the numerical model specimen was 2730 kg/m^3^, the minimum particle radius was 0.25 mm, and the ratio of the minimum particle radius to the maximum particle radius was 2.8. A total of 6156 particles of different sizes were generated, as shown in Fig. 2. The mesoscopic parameters of the parallel bond model mainly include effective modulus, parallel bond effective modulus, stiffness ratio, parallel bond stiffness ratio, tensile strength, cohesion, friction angle, and friction coefficient. Through the "trial and error method", first adjust the effective modulus and parallel bond effective modulus; secondly, adjust the parallel bond stiffness ratio; then adjust the normal bond strength and tangential bond strength; finally, adjust the friction angle and friction coefficient to determine the failure mode. The mesoscopic parameters are shown in Table 1.

**Table 1:** Parameters for detailed simulation of the assemblage.

Parameters	Symbol	Value
Density/(kg/m^3^)	*ρ*	2730
Porosity	*φ*	0.06
Elastic modulus/GPa	E*	7
Stiffness ratio	k*	1.5
Friction coefficient	*μ*	0.6
Parallel bond effective modulus/GPa	$\overline{E}^{*}$	7
Parallel bond stiffness ratio	$k^{*}$	1.3
Normal bond strength/MPa	$\overline{\sigma }_{c}$	15
Tangential bond strength/MPa	$\overline{c}$	16
Friction angle/°	$\overline{\phi }$	20

**Fig. 3:**
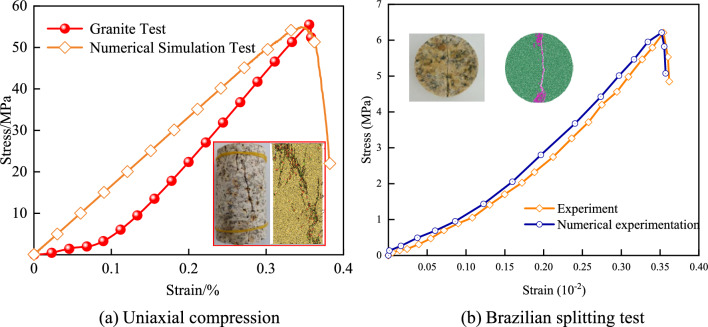
Comparison between laboratory test results and numerical simulation results.

The results of the granite uniaxial compression test and the numerical simulation test are shown in Fig. 3. Based on PFC particle flow, the compaction stage cannot be simulated because the particles in the initial model are homogeneously distributed. As shown in Table 2, the errors between the elastic modulus and peak strength obtained from the uniaxial compression tests on granite and those from the numerical simulations are relatively small. The peak stress, peak strain, and elastic modulus from the laboratory uniaxial compression tests are 55.5 MPa, 0.356 × 10^–2^, and 19.34 GPa, respectively; while those from the uniaxial compression numerical simulations are 54.83 MPa, 0.347 × 10^–2^, and 16.54 GPa, respectively. The errors between the two sets of results are 1.2%, 2.5%, and 14.14%, respectively. The failure modes observed in the granite experiments are similar to those in the numerical simulations, indicating that the mesoscopic parameters selected in this study are reasonable. Therefore, the mesoscopic parameters of the numerical model in Table 1 can be used to carry out uniaxial compression tests on pre-fabricated fractured granite to analyze the mechanical properties and energy damage evolution mechanism of granite.

**Table 2:** Comparison between indoor test results and numerical simulation results.

	Peak Stress	Peak Strain	Elastic Modulus
Laboratory Uniaxial Compression Test	55.5 MPa	0.356 × 10^–2^	19.34GPa
Numerical Uniaxial Compression Simulation	54.83 MPa	0.347 × 10^–2^	16.54GPa
Error	1.2%	2.5%	14.4%

The Brazilian disc splitting test on granite yielded a peak stress of 6.23 MPa, while the PFC numerical model produced a peak stress of 6.21 MPa, showing a negligible difference of only 0.3%. Similarly, the peak strain measured in the laboratory test was 0.34 × 10⁻^2^, compared to 0.35 × 10⁻^2^ in the PFC simulation, with a marginal deviation of 2.9%. Moreover, the macroscopic failure patterns observed in both physical and numerical tests exhibited strong consistency. As illustrated in Fig. 3, the tensile-to-compressive strength ratio of granite was approximately 1:8.9 in experimental tests and 1:8.8 in numerical simulations. These results demonstrate that the particle flow model not only effectively replicates the macroscopic mechanical behavior of granite but also accurately captures its compressive and tensile characteristics. This approach significantly enhances the reliability of analyzing granite’s damage evolution and failure mechanisms.

### Particle flow numerical test scheme

To analyze the effects of fracture inclination and length on the mechanical properties and energy damage characteristics of granite, a total of 21 working conditions were established for uniaxial compression numerical simulations. The centroid distances of the prefabricated fractures to the upper and lower surfaces are both 50 mm, the fracture width (*D*) is 1.5 mm, and the fracture lengths (*L*) are 10 mm, 20 mm, and 30 mm. The fracture inclinations (*α*) are 0°, 15°, 30°, 45°, 60°, 75°, and 90°, as detailed in Table 3.

**Table 3:** Scheme of numerical simulation.

Fracture Length/mm	Fracture Inclination/°						
	0	15	30	45	60	75	90
10	A1	A2	A3	A4	A5	A6	A7
20	B1	B2	B3	B4	B5	B6	B7
30	C1	C2	C3	C4	C5	C6	C7

## Analysis of numerical simulation test results

### Stress—strain curves

**Fig. 4:**
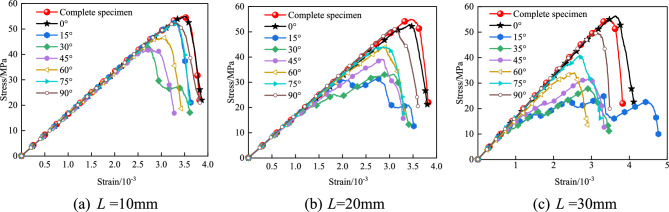
Granite stress–strain curve.

The stress–strain curves of granite with different fracture lengths and inclination angles during uniaxial compression are shown in Fig. 4. It can be seen from Fig. 4 that the granite specimens with different fracture lengths and inclination angles all have a linear elastic stage, a crack initiation stage, a crack propagation stage (yield stage), and a post-failure stage. For the specimens with *L* = 20 mm and *L* = 30 mm, a partial stress drop platform phenomenon appears, which is due to the evolution of macro cracks in the specimens during the continuous loading of the external load and is related to the shape of the pre-fabricated fractures. In the linear elastic stage, the specimen is in a recoverable deformation state, and no cracks are generated; in the crack initiation stage, under the continuous action of the load, the specimen begins to enter a plastic deformation state, and a small number of micro cracks are generated at both ends of the fracture; in the crack propagation stage (yield stage), the specimen enters an accelerated failure stage, and an obvious stress concentration phenomenon occurs. A large number of cracks initiate and propagate along the ends of the fracture and penetrate, and the strength of the specimen reaches the peak; in the post-failure stage, the stress tends to zero, indicating that the specimen still has a certain bearing capacity. The micro cracks expand and penetrate to form a macro failure surface. Selecting the fracture inclination angle *α* = 0° to analyze the failure process of granite under different fracture lengths, when L = 10 mm, the peak stress is 55 MPa; when *L* = 20 mm, the peak stress is 52.3 MPa; when *L* = 30 mm, the peak stress is 55.4 MPa, indicating that the influence of fracture length on the peak strength is weaker than that of the fracture inclination angle.

When *L* = 10 mm, with the change of the fracture inclination angle, the compressive strength of granite gradually decreases from 0° to 45°; and gradually increases from 45° to 90°. The peak strength of granite is the largest when the fracture inclination angle is 0°, and the lowest when the fracture inclination angle is 45°. When *L* = 20 mm, with the change of the fracture inclination angle, the compressive strength of granite gradually decreases from 0° to 15°; and gradually increases from 30° to 90°. The peak strength of granite is the largest when the fracture inclination angle is 0°, and the lowest when the fracture inclination angle is 15°. When *L* = 30 mm, the change law of the compressive strength of granite is the same as that when *L* = 20 mm. When the fracture inclination angle is the same, with the increase of the pre-fabricated fracture length, the compressive strength gradually decreases.

### Strength characteristics

**Fig. 5:**
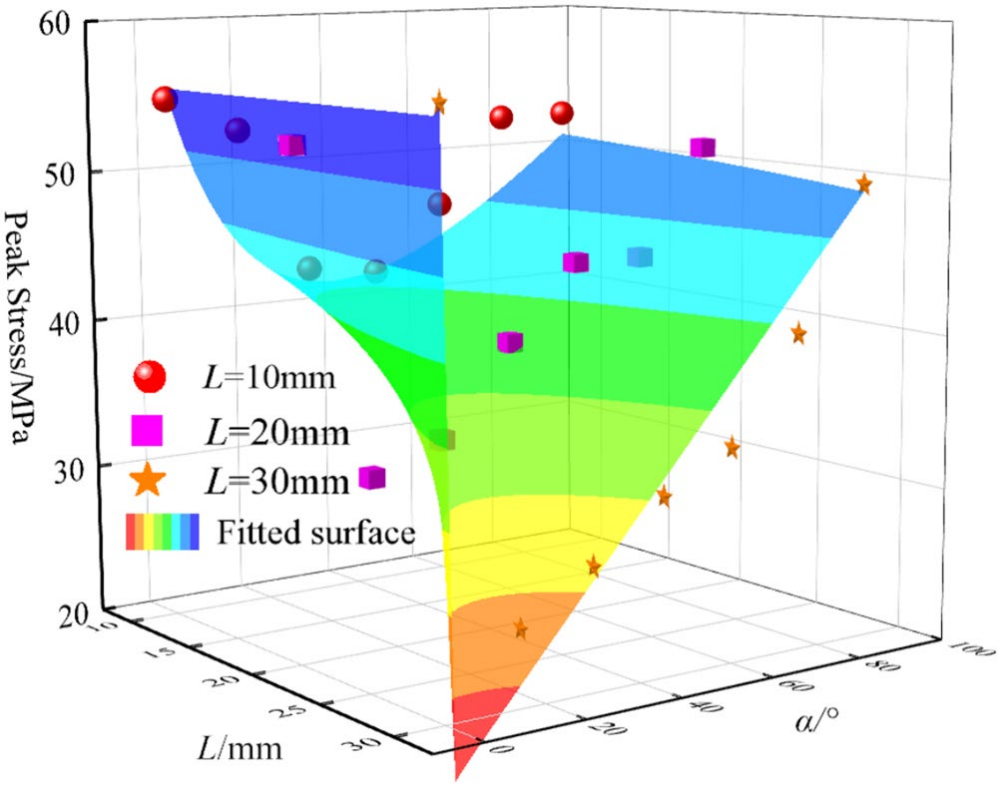
The peak stress of granite under different crack length and dip angle.

**Fig. 6:**
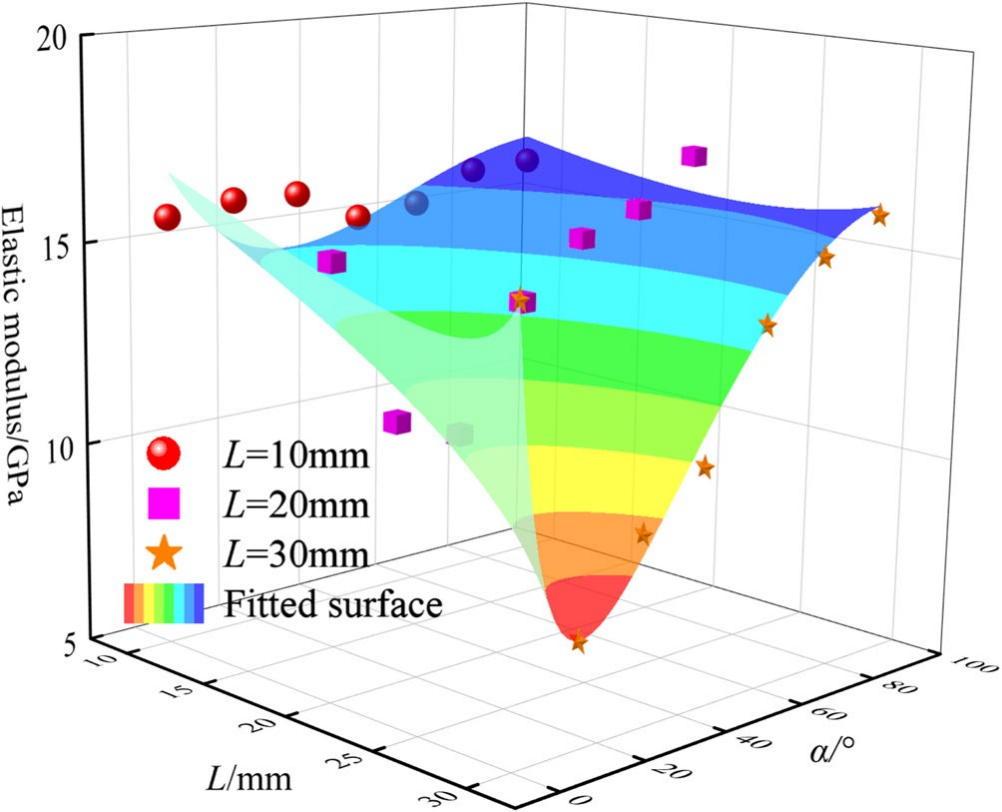
The elastic modulus of granite under different crack length and dip angle.

The peak stress and elastic modulus of granite with different fracture lengths and inclination angles under uniaxial compression tests are shown in Fig. 5 and Fig. 6. When *L* = 10 mm, the average peak stress of granite is 49.06 MPa, the peak stress of granite with a fracture inclination angle of 0° is 54.9 MPa, and the peak stress of granite with a fracture inclination angle of 45° is 42.07 MPa, with a peak strength difference of 23%; when *L* = 20 mm, the average peak stress is 42.17 MPa, and its strength is significantly lower than that of granite with a fracture length of 10 mm. The peak stress of granite with a fracture inclination angle of 0° is 52.93 MPa, and the peak stress of granite with a fracture inclination angle of 15° is 31.4 MPa, with a peak strength difference of 40.6%; when *L* = 30 mm, the average peak stress of granite is 37.82 MPa, the peak stress of granite with a fracture inclination angle of 0° is 56.35 MPa, and the peak stress of granite with a fracture inclination angle of 15° is 24.93 MPa, with a peak strength difference of 55.7%.

The results show that the larger the fracture length, the lower the peak strength of granite. A prediction model of the relationship between different fracture lengths, inclination angles, and peak stress was established, with a good fitting degree, *R*^2^ = 0.93 and high correlation:

1 $$\sigma { = }\frac{{{55}{\text{.61 - 1}}{.85}L{ + 0}{\text{.33}}\alpha { + 0}{\text{.0076}}\alpha^{{2}} }}{{{1 - 0}{\text{.03}}L{ + 0}{\text{.02}}\alpha }}$$

where *σ* is the peak stress, MPa; *L* is the fracture length, mm; *α* is the fracture length, °。

It can be observed from Fig. 6 that when the pre-fabricated fracture length is 10 mm, its average elastic modulus is higher than that of the pre-fabricated fracture lengths of 20 mm and 30 mm. When *L* = 10 mm, the average elastic modulus is 15.62 GPa, and the change of the elastic modulus of granite under different fracture inclination angles is small; when *L* = 20 mm, the average elastic modulus is 14.12 GPa, and the elastic modulus reaches the maximum value of 16.75 GPa when the fracture inclination angle is 90°, and the minimum value of 10.78 GPa when the fracture inclination angle is 30°, indicating that the elastic modulus is affected by the fracture inclination angle; when *L* = 30 mm, the average elastic modulus is 12.67 GPa, and the elastic modulus reaches the maximum value of 16.02 GPa when the fracture inclination angle is 90°, and the minimum value of 7.55 GPa when the fracture inclination angle is 15°. The research shows that with the increase of the pre-fabricated fracture length, its elastic modulus gradually decreases, and the load-bearing performance of the specimen gradually weakens.

A prediction model of the relationship between different fracture lengths, inclination angles and elastic modulus was established, with a relatively high fitting degree, *R*^2^ = 0.85;

2 $$E = \frac{{{18}{\text{.67 - 0}}{.768}L + {0}{\text{.006}}L^{2} { - 0}{\text{.027}}\alpha { + 0}{\text{.00286}}\alpha^{2} { - 0}{\text{.0000149}}\alpha^{3} }}{{1{ - 0}{\text{.03}}L + {0}{\text{.0067}}\alpha }}$$

where *E* is the elastic modulus, GPa; *L* is the fracture length, mm; *α* is the fracture inclination angle, °.

### Microcrack evolution law

**Fig. 7:**
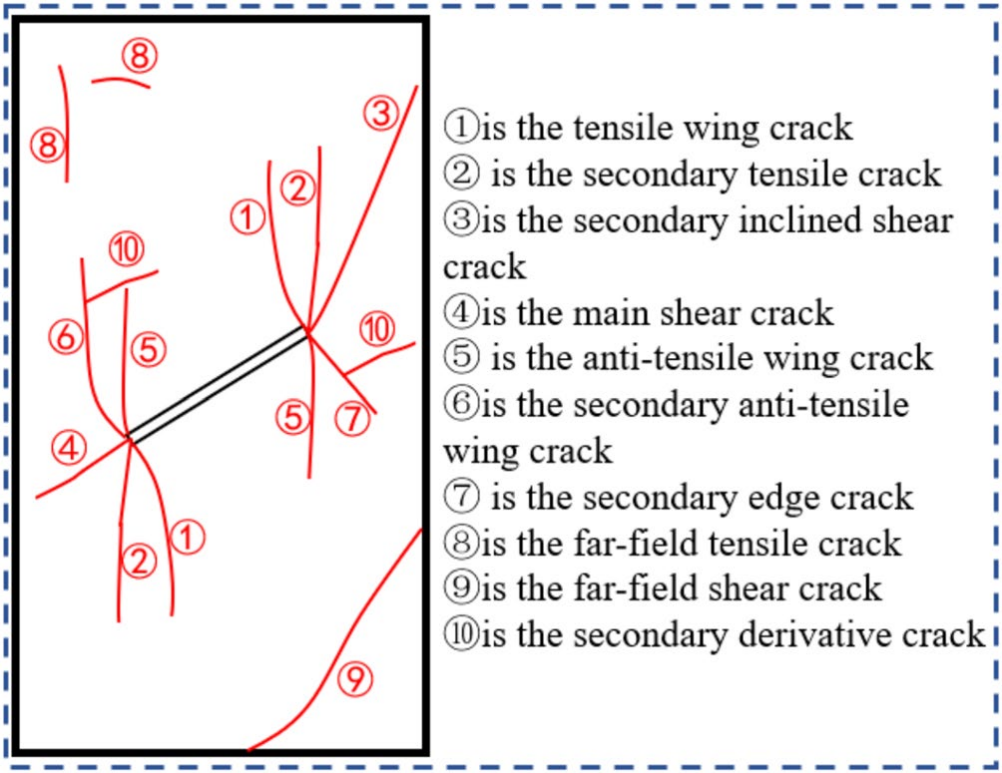
Classification of different types of cracks in precast fissure rocks.

During the damage and failure process of rock, not only are macroscopic, visible through-going fractures formed, but a large number of randomly distributed microcrack networks also develop within the rock mass. Studies on the failure characteristics of rocks with prefabricated fractures indicate that crack initiation and propagation mainly originate from the stress concentration zones at the tips of prefabricated fractures, and the evolution process is significantly correlated with the geometric characteristics of the prefabricated fractures (including fracture inclination, length, aspect ratio, etc.). Based on an in-depth analysis of fracture geometry, spatial distribution angles, and propagation mechanisms, and in combination with the latest research findings on crack propagation both domestically and internationally^25,26^, this study systematically identifies and classifies ten characteristic types of cracks generated during the damage and failure process of prefabricated geometric fractures through experimental observation and numerical simulation, as shown in Fig. 7. These include: (1) tensile wing cracks; (2) secondary tensile cracks; (3) secondary inclined shear cracks; (4) primary shear cracks; (5) anti-tensile wing cracks; (6) secondary anti-tensile wing cracks; (7) secondary edge cracks; (8) far-field tensile cracks; (9) far-field shear cracks; and (10) secondary derivative cracks. It is noteworthy that distinct tensile stress concentration zones and compressive stress shielding zones are formed around the prefabricated fractures, and this stress differentiation directly leads to the initiation of microcracks. When the maximum tensile stress in the specimen exceeds its tensile strength threshold, microcracks begin to nucleate and propagate preferentially in the stress concentration regions. The specific manifestations of this dynamic evolution process are detailed in the experimental results shown in Fig. 7.

**Fig. 8:**
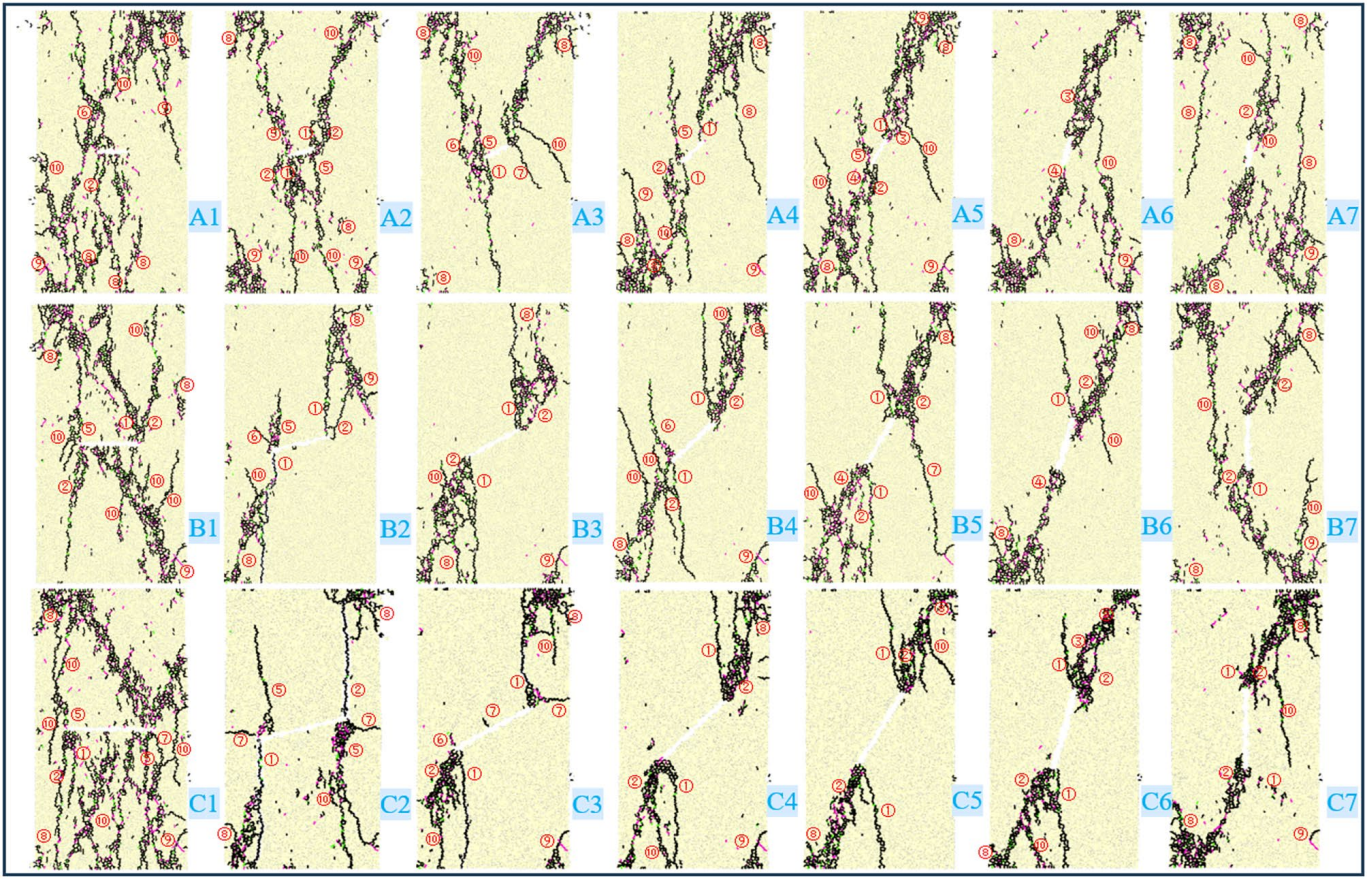
Uniaxial compression failure modes of granite under different crack lengths and dip angles.

The failure modes of granite under uniaxial compression tests with different fracture lengths and inclination angles are shown in Fig. 8. The pink cracks represent shear-compression cracks, the green cracks represent shear-tension cracks, and the black cracks represent tensile cracks. When the fracture inclination angle is 0°, tensile wing cracks, secondary tensile cracks, far-field tensile cracks, secondary derivative cracks, and anti-tensile wing cracks are mainly derived in the fractured granite. The failure mode is a through shear-tension composite failure. Cracks first initiate from the upper left corner, and then tensile wing cracks and secondary tensile cracks develop at both ends of the fracture. From the upper left through the right end of the pre-fabricated fracture to the lower right, a macro failure surface is formed. The larger the pre-fabricated fracture, the more microcracks and the more severe the failure. When the fracture inclination angle is 15°, tensile wing cracks, secondary tensile cracks, anti-tensile wing cracks, far-field tensile cracks, far-field shear cracks, secondary derivative cracks, and secondary edge cracks are derived, resulting in a conjugate shear-tension failure mode. It can be observed from C2 that there is a macro fracture zone, which is due to the joint influence of the fracture inclination angle and length on the crack initiation, propagation, and penetration. Microcracks first develop from the upper right and lower left of the specimen, and microcracks are derived from the ends of the pre-fabricated fracture under the action of the load. Finally, a conjugate macro failure is formed from the upper right through the fracture ends to the lower left. When the fracture inclination angles are 30°, 45°, 60°, and 75°, tensile wing cracks, secondary tensile cracks, secondary inclined shear cracks, main shear cracks, anti-tensile wing cracks, far-field tensile cracks, far-field shear cracks, and secondary derivative cracks are mainly derived. The penetration of cracks mainly develops from both ends of the pre-fabricated crack, and wing cracks are evolved in the specimen. Some wing cracks are parallel to the pre-fabricated fracture, and the failure modes are anti-wing crack shear failure and wing crack tension failure. When the fracture inclination angle is 90°, tensile wing cracks, secondary tensile cracks, far-field tensile cracks, far-field shear cracks, and secondary derivative cracks are generated in the specimen. Microcracks develop from the upper right and lower left of the specimen and finally penetrate the pre-fabricated fracture to form an approximately “triangular” failure body, and the failure mode is shear-tension failure. In summary, the failure of the specimen is mainly shear with tension as an auxiliary. The number of microcrack developments is affected by the length of the pre-fabricated fracture, and the crack initiation direction is greatly affected by the fracture inclination angle.

## Influence of fracture inclination angle and length on the energy evolution law of granite

The conversion and loss of energy are the essence of rock failure and correspond to the closure of microcracks, the propagation and penetration of new microcracks, and the formation of main cracks. During this process, the rock strength decreases, causing the rock to eventually lose its bearing capacity. With the change of stress state and the development of deformation, the energy state of the rock is constantly changing, and the micro-defects in the rock evolve from disordered distribution to orderly development, forming macro cracks. Eventually, macro cracks accumulate in a certain direction, leading to overall instability. Therefore, studying the energy evolution law of rocks is of great significance for explaining the mechanical characteristics and failure mechanism of rocks under stress.

### Energy calculation principle

The deformation and failure process of rock is not merely a mechanical behavior, but essentially an instability evolution process driven by energy. This process is accompanied by energy input, accumulation, dissipation, and release, reflecting the dynamic adjustment of the internal state of the rock under external loading^27^. During loading, the rock stores energy through elastic deformation on the one hand, and continuously dissipates energy due to the generation and development of microcracks on the other; these two processes occur almost simultaneously and jointly govern the structural evolution.

Based on the fundamental principles of thermodynamics, and assuming a closed system with no heat exchange, the work done by external forces can be decomposed into elastic energy and dissipated energy, corresponding to the reversible deformation and irreversible damage processes of the material, respectively^28–32^. Among them, the elastic energy density *U*_e_ reflects the energy storage capacity of the rock, while the dissipated energy density *U*_d_ characterizes the degree of damage evolution. The calculation formulas are as follows.

3 $$U = U_{{\text{e}}} + U_{{\text{d}}}$$

4 $$U = \int_{0}^{{\varepsilon_{1} }} {\sigma_{1} } {\text{d}}\varepsilon_{1}$$

5 $$U_{{\text{e}}} = \frac{1}{2}\sigma_{1} \varepsilon_{{1}}^{{\text{e}}} = \frac{{\sigma_{1}^{2} }}{2E}$$

where *U* is the total energy of the rock sample, J·cm^−3^; *U*_d_ is the dissipated energy of the rock sample, J·cm^−3^, which is caused by the plastic strain and elastic damage of the material and is irreversible during loading and unloading; *U*_e_ is the elastic energy of the rock sample, which is stored in the material in the form of internal energy during loading and released in the form of recoverable strain during unloading, J·cm^−3^.

The energy density is calculated by the graphical integration method, and the total energy *U* is calculated as follows:

6 $$U = \sum\limits_{i = 1}^{n} {\frac{{(\sigma_{1}^{i} + \sigma_{1}^{i + 1} )(\varepsilon_{1}^{i} + \varepsilon_{1}^{i + 1} )}}{2}}$$

where $\sigma_{1}^{i}$ is the stress at any point on the axial stress—strain curve of the rock sample, MPa; $\varepsilon_{1}^{i}$ is the strain at any point on the stress—strain curve.

### Energy evolution law of geometric fractured granite

**Fig. 9:**
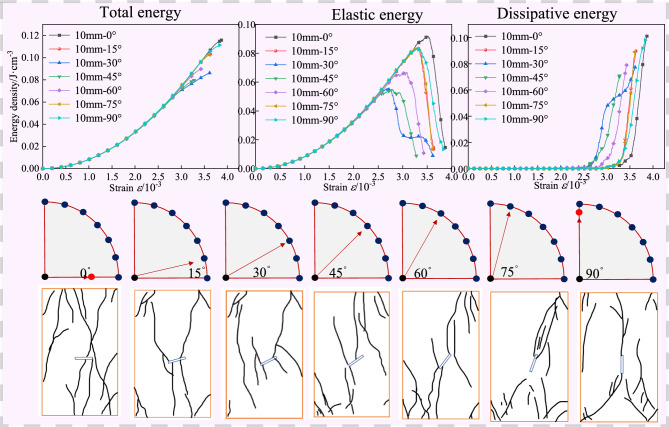
The internal energy evolution of granite samples with different dip angles under the fracture length of 10 mm.

**Fig. 10:**
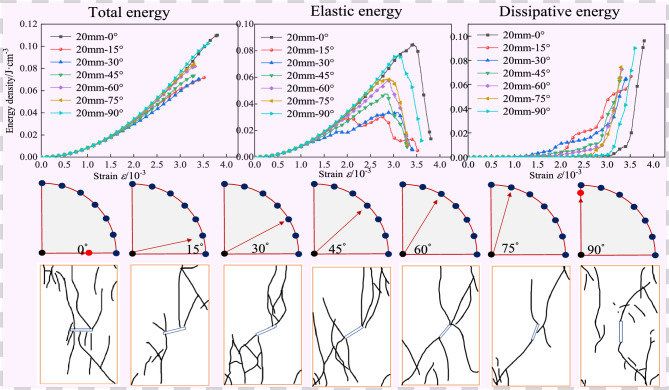
The internal energy evolution of granite samples with different dip angles under the fracture length of 20 mm.

To explore the influence of fracture inclination angle and fracture length on the energy and microcrack evolution laws of geometric fractured granite during uniaxial compression, the energies under different fracture inclination angles and fracture lengths are calculated and the evolution curves are drawn. The internal energy evolution curves of specimens with a fracture length of 10 mm are shown in Fig. 9, where the upper, middle, and lower parts of Fig. 9 correspond to the energy evolution curve, fracture inclination, and the sketch of specimen failure, respectively. As shown in Fig. 10, under different fracture inclination angles, the total energy—strain curve of the specimen shows a semi-parabolic growth trend, and the elastic energy also shows a similar semi-parabolic increase trend before reaching the peak. After the peak strain, the elastic strain energy decreases rapidly, while the dissipated energy—strain curve is relatively stable in the initial loading stage, with almost no obvious fluctuation, indicating that the specimen is not damaged in the initial loading stage. With the continuation of loading, especially after reaching the peak, the dissipated energy increases significantly, indicating the complete failure of the specimen and the complete release of internal energy. It can be observed that the role of dissipated energy is relatively insignificant during the initial loading stage, with elastic energy being the dominant form. When a substantial amount of elastic energy accumulates, crack propagation occurs along the periphery of the pre-existing fissures, resulting in increased crack accumulation^33,34^. Following crack coalescence and further development, dissipated energy becomes the predominant energy form.

When the fracture inclination angle is 30°, the total energy, elastic energy, and dissipated energy corresponding to the peak stress reach the minimum values, which are 0.0592, 0.05567, and 0.00353, respectively; when the fracture inclination angle is 0°, the total energy and elastic energy corresponding to the peak stress reach the maximum values, which are 0.10012 and 0.09194, respectively; when the fracture inclination angle is 60°, the dissipated energy corresponding to the peak stress reaches the maximum value of 0.01065. The total energy and elastic energy both increase by 1.7 times, and the dissipated energy increases by 3 times. The elastic strain energy at the peak point can reflect the intensity of energy absorption of the specimen during uniaxial compression. The larger the elastic strain energy at the peak point, the more energy the specimen absorbs, and the greater the possibility of deformation and fracture. The greater the released energy, the more macro cracks are generated in the specimen, and the greater the degree of specimen failure.

The research shows that when the fracture length is 10 mm, during the process of increasing the fracture inclination angle from 0° to 90°, the specimen is more likely to fracture and fail when the inclination angle is 0°, and the specimen is less likely to fracture and fail when the inclination angle is 30°, and at this time, the number of macro cracks is the smallest and the degree of failure is the smallest; when the inclination angle is 60°, the energy release of the specimen is the largest, and the number of macro cracks reaches the maximum, and the degree of failure reaches the maximum.

The internal energy evolution curve of the specimen with a fracture length of 20 mm is shown in Fig. 10. When the fracture inclination angle is 15°, the total energy and elastic energy corresponding to the peak stress reach the minimum values, which are 0.05513 and 0.0304, respectively; when the fracture inclination angle is 90°, the dissipated energy corresponding to the peak stress reaches the minimum value of 0.0048. When the fracture inclination angle is 0°, the total energy and elastic energy corresponding to the peak stress reach the maximum values, which are 0.0945 and 0.0857, respectively; when the fracture inclination angle is 30°, the dissipated energy corresponding to the peak stress reaches the maximum value of 0.0279. Among them, the total energy increases by 1.7 times, the elastic energy increases by 2.8 times, and the dissipated energy increases by 5.7 times. The research shows that when the fracture length is 20 mm, during the process of increasing the fracture inclination angle from 0° to 90°, the specimen is more likely to fracture and fail when the inclination angle is 0°, and the specimen is less likely to fracture and fail when the inclination angle is 15°; when the inclination angle is 30°, the energy release of the specimen is the largest, and the number of macro cracks reaches the maximum, and the degree of failure reaches the maximum; when the inclination angle is 90°, the number of macro cracks is the least and the degree of failure is the smallest.

**Fig. 11:**
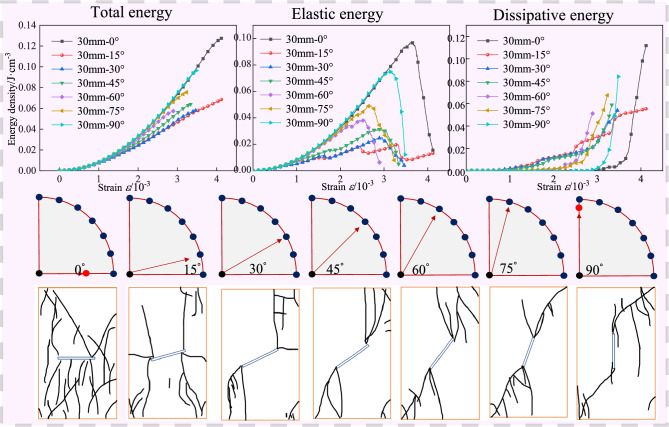
The internal energy evolution of granite samples with different dip angles under the fracture length of 30 mm.

The internal energy evolution curve of the specimen with a fracture length of 30 mm is shown in Fig. 11. When the fracture inclination angle is 30°, the total energy corresponding to the peak stress reaches the minimum value of 0.04705. When the fracture inclination angle is 15°, the elastic energy corresponding to the peak stress reaches the minimum value of 0.01981. When the fracture inclination angle is 90°, the dissipated energy corresponding to the peak stress reaches the minimum value of 0.00702; when the fracture inclination angle is 0°, the total energy and elastic energy corresponding to the peak stress reach the maximum values, which are 0.10684 and 0.09747, respectively; when the fracture inclination angle is 15°, the dissipated energy corresponding to the peak stress reaches the maximum value of 0.03407. Among them, the total energy increases by 2.3 times, the elastic energy increases by 4.9 times, and the dissipated energy increases by 4.9 times.

In summary, when the fracture length is 30 mm, during the process of increasing the fracture inclination angle from 0° to 90°, the specimen is more likely to fracture and fail when the inclination angle is 0°, and the specimen has the largest energy release when the inclination angle is 15°, and the number of macro cracks reaches the maximum, and the degree of failure reaches the maximum; when the inclination angle is 90°, the number of macro cracks is the least and the degree of failure is the smallest. By comparing Fig. 9, Fig. 10, and Fig. 11, it is found that under different fracture lengths, the angles at which the specimen is most likely to fracture and the angles at which the specimen has the largest degree of failure are different. However, when the inclination angle is 0°, the specimen is more likely to fracture and fail. With the increase of the fracture length, the inclination angles corresponding to the maximum number of macro cracks and the largest degree of failure of the specimen decrease instead. For overall analysis, the change trend can be divided into four stages, namely: compaction stage, elastic stage, yield stage, and failure stage. The peak stress point is not only the critical point of rock strength loss and macro fracture surface connection but also the critical moment of elastic energy and dissipated energy conversion^35,36^. With the increase of the fracture inclination angle, the total energy continues to rise, and the elastic energy and dissipated energy show a trend of first increasing and then decreasing. Specifically, in the initial loading stage, most of the input energy is converted into elastic energy stored in the specimen, and only a small part of the energy is dissipated. When the stress reaches the peak, the rock enters a high-energy unstable state, and energy begins to be released, and the release rate rises sharply. After entering the yield stage, part of the input energy is dissipated with the closure and propagation of cracks, and the rest of the input energy is still stored in the form of released elastic energy. Once the released elastic energy stored in the specimen exceeds the limit value, the specimen will break due to the release of elastic energy^37^.

### Strain energy rate law analysis

As mentioned above, there are obvious differences in the elastic energy and dissipated energy sizes in the crack initiation and crack damage stages under different working conditions, which also indicates that there are large differences in the damage degrees. However, it is not comprehensive enough to study the damage degree only from one aspect of elastic energy or dissipated energy. Therefore, to further understand the difference in rock energy balance, the concept of strain energy rate *YU* is proposed and explained as follows^38^.

**Fig. 12:**
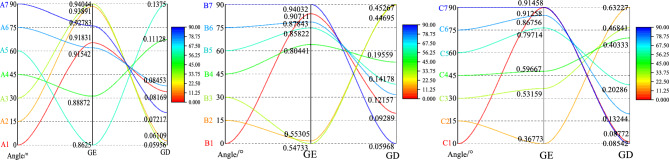
The evolution of energy release rate and energy dissipation rate in granite samples with different dip angle.

Define *U*_*e*_*/U* as the energy release rate, denoted as *GE*, which can represent the ability to absorb elastic strain energy; *U*_*d*_*/U* is defined as the energy dissipation rate, denoted as *GD*, which represents the variables of strain energy accumulation and dissipation. *GE* and *GD* can effectively reveal the essence of rock deformation and cracking mechanisms. Define *GE/GD* as the strain energy rate, denoted as *YU*. The change trends of different fracture lengths and inclination angles are drawn as shown in Fig. 12.

It can be seen from Fig. 12 that when the fracture length is 10 mm, *GE* reaches the maximum when the fracture inclination angle is 30° and the minimum when the fracture inclination angle is 60°. *GD* reaches the maximum when the fracture inclination angle is 60° and the minimum when the fracture inclination angle is 30°; when the fracture length is 20 mm, *GE* reaches the maximum when the fracture inclination angle is 90° and the minimum when the fracture inclination angle is 30°. *GD* reaches the maximum when the fracture inclination angle is 30° and the minimum when the fracture inclination angle is 90°; when the fracture length is 30 mm, *GE* reaches the maximum when the fracture inclination angle is 90° and the minimum when the fracture inclination angle is 15°. *GD* reaches the maximum when the fracture inclination angle is 15° and the minimum when the fracture inclination angle is 90°. This change trend shows that the fracture length and fracture inclination angle have an obvious nonlinear coupling effect on *GE* and *GD*. With the increase of the fracture length, the position of the maximum value of *GE* gradually shifts to a larger fracture inclination angle, and the position of the maximum value of *GD* gradually shifts to a smaller fracture inclination angle, which reflects the influence of fractures with different geometric parameters on the energy storage and release mechanisms of the specimen. In addition, the energy characteristic curves under different fracture parameters show a rapid change near the critical point, indicating that the crack propagation and failure process inside the specimen has a high degree of directionality and geometric sensitivity.

**Fig. 13:**
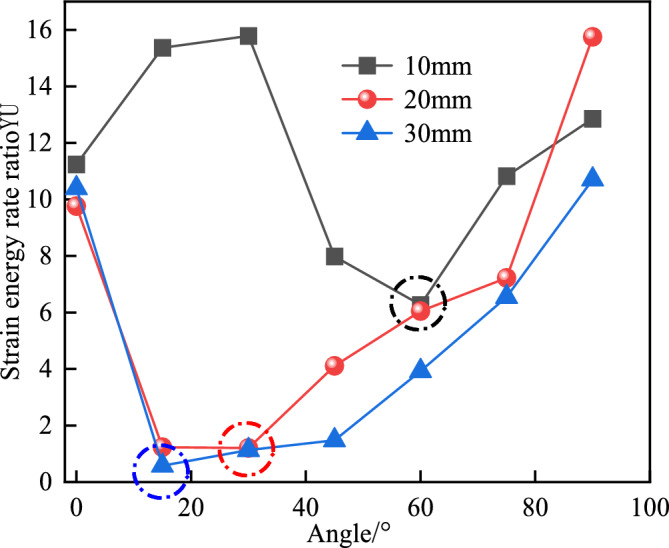
The strain energy rate of granite samples under different working conditions.

The research shows that under the same fracture length, the maximum and minimum values of *GE* and *GD* are exactly opposite, which is in contrast to the above-mentioned conversion of external work into elastic strain energy and dissipated energy according to the first and second laws of thermodynamics. Although *GE* and *GD* reach their maximum and minimum values under different conditions of fracture length and inclination angle, it also reflects that there is an optimal length and inclination angle under different fracture inclination angles and lengths, so that the damage degree of the granite specimen is the smallest. To further analyze its evolution trend, the strain energy rate *YU* under different working conditions is drawn as shown in Fig. 13.

It can be found from Fig. 13 that when the fracture length is 10 mm, it shows an"increase—decrease—increase"evolution trend, while when the fracture length is 20 mm and 30 mm, it shows a"decrease—slow increase—rapid increase"trend. When the fracture length is 10 mm and the angle is 60°, the minimum value of *YU* is 6.27; when the fracture length is 20 mm and the angle is 30°, the minimum value of *YU* is 1.21; when the fracture length is 30 mm and the angle is 15°, the minimum value of *YU* is 0.58. The smaller the *YU* value, the smaller the difference between the accumulation of elastic energy and the release of dissipated energy, the greater the released energy, the more the number of macro cracks generated in the specimen, the greater the degree of specimen failure, and the more conducive to the generation of cracks, which is consistent with the conclusions drawn above. The research shows that the strength and failure mode of geometric fractured granite are important indicators for engineering design and construction. Especially in key engineering links such as roadway support and roof cutting and pressure relief, accurately evaluating the mechanical properties and failure characteristics of rocks is crucial for ensuring the safety and stability of the project.

## Construction of the constitutive model

The essence of rock damage evolution is the absorption and release of energy. To further investigate the influence of different fracture lengths and inclinations on the energy of granite, a damage constitutive model is constructed to characterize their relationship.

According to J. Lemaitre’s equivalent strain assumption, the nominal stress and effective stress of granite satisfy the following relationships^39,40^:

7 $$F = \sigma A = \sigma^{\prime}A^{\prime}$$

8 $$\sigma = \frac{{A^{\prime}}}{A}\sigma^{\prime}$$

where *F* is the load applied to the specimen (N);σ is the nominal stress of the specimen under uniaxial loading; $\sigma^{\prime}$ is the effective stress of the specimen under uniaxial loading (MPa); *A* is the nominal bearing area of the specimen under uniaxial loading; $A^{\prime}$ is the nominal bearing area of the specimen under uniaxial loading (mm^2^).

Referring to the definition of the damage factor in^37^, the internal damage expression of uniaxial compression specimens under different loading rates is given by formula (9):

9 $$D = \frac{{A - A^{\prime}}}{A} = 1 - \frac{{A^{\prime}}}{A}$$

where *D* is the damage factor of the specimen under uniaxial loading, and *D* ∈ [0, 1].

According to Eqs. (8) and (9), Eq. (10) is obtained:

10 $$\sigma = \sigma^{\prime}(1 - D)$$

Based on the strain compatibility deformation assumption for specimens under uniaxial loading, the effective stress satisfies the linear Hooke’s law. According to Eq. (10), the constitutive relationship for the stress–strain curve of specimens under uniaxial loading is given by Eq. (11):

11 $$\sigma = E\varepsilon (1 - D)$$

According to the principles of statistical damage mechanics, and considering the correlation between the damage factor and energy dissipation of specimens under uniaxial compression, the specimen under uniaxial compression can be regarded as composed of countless micro-units. The damage factor *D* of the specimen can be expressed as the ratio of the number of failed micro-units to the total number of micro-units. Therefore, it is assumed that the probability of failure of micro-units in the specimen under uniaxial compression and the dissipated energy satisfy the generalized Weibull statistical probability density relationship. With the increase of dissipated energy, the number of failed micro-units in granite under uniaxial compression is:

12 $$N = M\left\{ {1 - \exp \left( {\frac{{p_{1} U_{d} + p_{2} U_{d}^{2} }}{{1 + p_{3} U_{d}^{2} }}} \right)} \right\}$$

where *N* and *M* are the number of damaged micro-units and the total number of micro-units in concrete, respectively; $p_{1}$, $p_{2}$, $p_{3}$, and $p_{4}$ are the parameters of the generalized Weibull distribution. Thus, the damage factor can be expressed as:

13 $$D = \frac{N}{M} = 1 - \exp \left( {\frac{{p_{1} U_{d} + p_{2} U_{d}^{2} }}{{1 + p_{3} U_{d}^{2} }}} \right)$$

According to the above formulas, the constitutive relationship for energy dissipation damage of specimens under uniaxial compression with different FRP cloth constraints can be obtained.

14 $$\sigma = E\varepsilon \cdot \exp \frac{{p_{1} U_{d} + p_{2} U_{d}^{2} }}{{1 + p_{3} U_{d}^{2} }}$$

The above is the damage constitutive model, in which there are three variable parameters: $p_{1}$, $p_{2}$, $p_{3}$. By substituting the elastic modulus, stress–strain curve, and dissipated energy of each group of models into the above formula, and then performing nonlinear fitting, the values of $p_{1}$, $p_{2}$, $p_{3}$ are obtained. The values of $p_{1}$, $p_{2}$, $p_{3}$ under different working conditions are listed in the following Tables 4, 5, and 6.

**Table 4:** Variable parameters for a fracture length of 10 mm.

Category	10–0°	10–15°	10–30°	10–45°	10–60°	10–75°	10–90°
p_1_	−8.9026	−0.7098	3.5439	13.4410	7.8927	8.2356	7.9548
p_2_	3.3524	4.1099	11.4077	30.1295	1.7295	0.0401	1.6801
p_3_	0.1563	0.2976	0.3224	2.4295	−0.0846	−0.1711	0.1422

**Table 5:** Variable parameters for a fracture length of 20 mm.

Category	20–0°	20–15°	20–30°	20–45°	20–60°	20–75°	20–90°
p_1_	1.2257	−5.6129	1.5776	13.7335	−0.0023	−3.5645	5.4734
p_2_	−0.3591	9.3118	4.9478	0.8807	2.3543	0.6355	−1.2108
p_3_	−0.0791	0.3903	0.3201	0.2936	−0.2427	0.0049	−0.1939

**Table 6:** Variable parameters for a fracture length of 30 mm.

Category	30–0°	30–15°	30–30°	30–45°	30–60°	30–75°	30–90°
p_1_	24.9948	−11.7309	10.8225	0.3530	16.5445	4.7535	4.5169
p_2_	−0.1373	5.2403	0.3190	0.5310	1.9832	7.9129	21.4659
p_3_	−1.0470	−0.5520	0.1907	−0.0019	0.4558	0.1334	1.5330

The parameters $p_{1}$, $p_{2}$, $p_{3}$ are further modified under different fracture lengths and inclinations, resulting in the following formula.

15 $$p_{1} = \frac{{0.278 - 10.359L + 0.168\alpha - 14.164\alpha^{2} + 1.343\alpha^{3} }}{{1 - 0.010L - 21.762L^{2} + 3.037L^{3} - 0.077\alpha }}$$

16 $$p_{2} = \frac{15.2963 - 2.5715L + 0.2332\alpha }{{1 - 0.022L - 0.2528L^{2} + 0.0088L^{3} + 0.8390\alpha - 0.0109\alpha^{2} }}$$

17 $$p_{3} = \frac{{0.1120 - 0.0038L - 0.0045\alpha + 5.4285\alpha^{2} }}{{1 - 0.033L - 0.0311\alpha + 0.0004\alpha^{2} }}$$

**Fig. 14:**
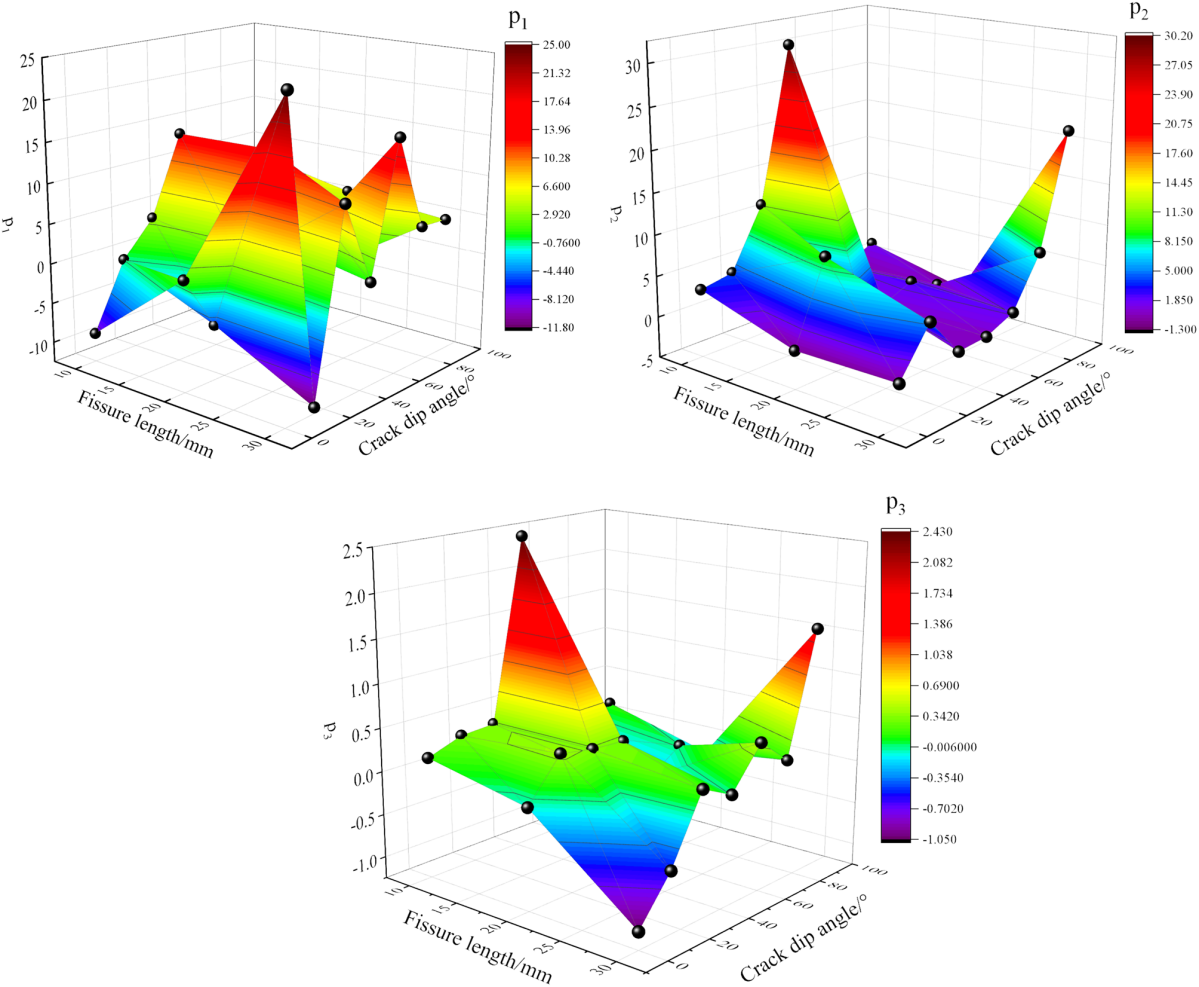
Relationship between Weibull distribution parameters and independent variate.

The three-dimensional surface fitting of the Weibull distribution parameters of granite under different fracture lengths and inclinations is shown in Fig. 14.

As shown in Fig. 14, the three-dimensional fitting surface formed by the Weibull distribution parameters $p_{1}$, $p_{2}$, $p_{3}$ of granite under different fracture lengths and inclinations exhibits a “paper crane” shape at different angles. Although the fluctuations of the parameters vary, $p_{1}$ fluctuates more significantly, while $p_{2}$ and $p_{3}$ are relatively uniform overall. However, all data points lie on the fitting surface, and the fitting coefficient *R*^2^ = 0.99, indicating a high degree of fit.

**Fig. 15:**
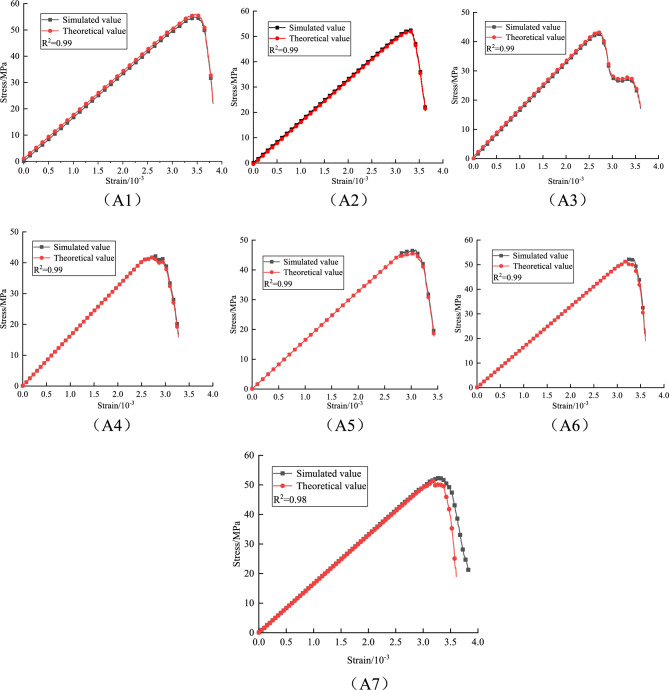
Validation of the intrinsic model at 10 mm fracture length.

**Fig. 16:**
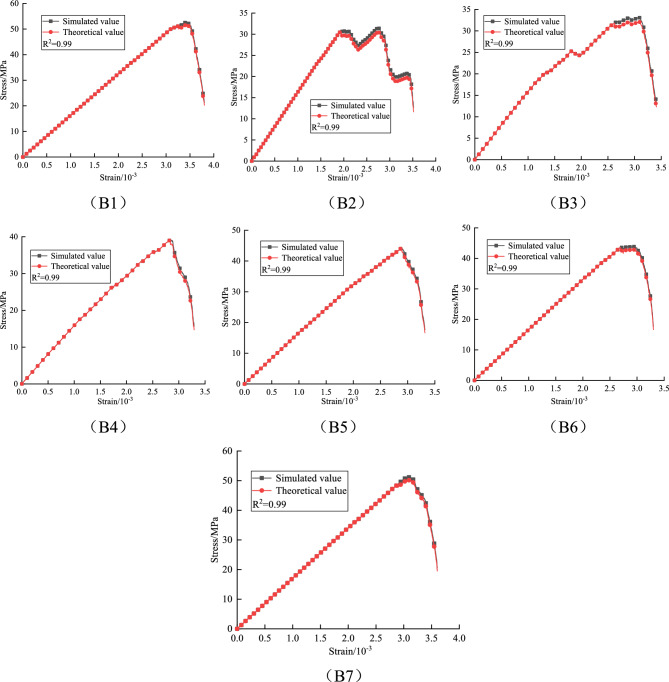
Validation of the intrinsic model at 20 mm fracture length.

**Fig. 17:**
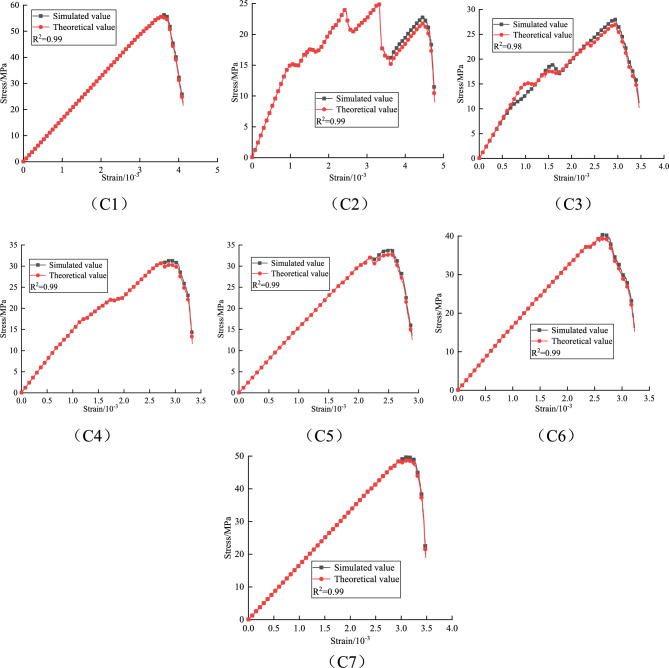
Validation of the intrinsic model at 30 mm fracture length.

Based on the above damage constitutive model, the theoretical values of granite under different fracture lengths and inclinations are compared with the stress–strain curves obtained from experiments, as follows:

A comparative analysis of the stress–strain curves in Figs. 15, 16, and 17 shows that the damage evolution constitutive equation can well fit the stress–strain curves of granite under different fracture lengths and inclinations. The various stages can be clearly distinguished from the fitted curves, and most of the errors occur near the peak or in regions of fluctuation. This is because, near the peak, the strain changes very rapidly and the stress monitoring values are subject to corresponding fluctuations, which affect the values of the fitting parameters. Overall, the theoretical model predictions are highly consistent with the numerical simulation data. This damage constitutive model effectively characterizes the pre-peak strengthening effect and post-peak residual weakening behavior of granite with different fracture lengths and inclinations, and systematically reveals the dynamic evolution mechanism of damage accumulation in fractured granite during unloading.

## Conclusions

(1) Relationship Between Mechanical Properties of Granite and Crack Parameters: The compressive strength and elastic modulus initially decrease and then increase with the increase of the prefabricated crack inclination angle (0° ~ 90°), reaching the lowest point at 15°. The failure mode is primarily shear with tension as a secondary factor. Crack propagation is influenced by the prefabricated length, while the crack initiation direction is mainly controlled by the inclination angle.

(2) Crack Propagation Law and Energy Evolution Mechanism: Failure is most likely to occur at an inclination angle of 0°. However, as the crack length increases, the inclination angles corresponding to the maximum number of macroscopic cracks and the highest degree of damage decrease sequentially (60° → 30° → 15°). Energy evolution consists of four stages: input, accumulation, dissipation, and release. Before the peak, elastic energy storage dominates, while after the peak, a sharp release of elastic energy leads to failure.

(3) Correlation Between Energy Parameters and Damage: Under the same crack length, the extreme values of elastic energy storage (GE) and dissipated energy (GD) exhibit an inverse relationship. A smaller YU value corresponds to greater released energy, more macroscopic cracks, higher damage levels, and more favorable conditions for crack generation.

(4) Validation of the Damage Constitutive Model: The established damage model, based on dissipated energy density, shows excellent agreement with experimental curves. It systematically reveals the dynamic evolution mechanism of damage accumulation in fractured granite during unloading and effectively characterizes the mechanical response under different crack parameters.

## Data Availability

The datasets used and analysed during the current study available from the corresponding author on reasonable request.
